# Appraisal of antivenom production in public laboratories in Latin America during the first semester of 2020: The impact of COVID-19

**DOI:** 10.1371/journal.pntd.0009469

**Published:** 2021-06-17

**Authors:** José María Gutiérrez, Larissa Zanette, Marco Antonio Natal Vigilato, Julio Cesar Augusto Pompei, Diogo Martins, Hui Wen Fan, the Latin American Network of Public Antivenom Manufacturing Laboratories (RELAPA)

**Affiliations:** 1 Instituto Clodomiro Picado, Facultad de Microbiología, Universidad de Costa Rica, San José, Costa Rica; 2 Centro Panamericano de Fiebre Aftosa, Organización Panamericana de la Salud, Rio de Janeiro, Brazil; 3 Wellcome Trust, London, United Kingdom; 4 Instituto Butantan, São Paulo, Brazil; Texas A&M University Kingsville, UNITED STATES

One of the 4 pillars of the World Health Organization (WHO) strategy for the prevention and control of snake bite envenomings is to ensure safe and effective pharmacotherapeutic treatments [1]. The mainstay in the pharmacotherapy of these envenomings, as well as of envenomings by scorpions and spiders, is the timely administration of safe and effective antivenoms [2,3]. Antivenoms are composed of immunoglobulins, or immunoglobulin fragments, purified from the plasma of animals, usually horses, immunized with venoms. Currently, there is an urgent need to improve antivenom availability, accessibility, and affordability on a global basis, particularly for use in sub-Saharan Africa, Asia, and Latin America [1].

There is a long tradition in snake, scorpion, spider, and, more recently, caterpillar antivenoms production in Latin America, especially centered in public manufacturing laboratories in Argentina, Brazil, Peru, Bolivia, Ecuador, Colombia, Venezuela, Costa Rica, and Mexico [4–6]. A network of public laboratories devoted to the production and quality control of antivenoms was established in this region in the last decade [4,5], which has recently led to the creation of the Latin American Network of Public Antivenom Manufacturing Laboratories (RELAPA, *Red Latinoamericana de Laboratorios Públicos Productores de Antivenenos*) [6]. RELAPA aims at consolidating governance mechanisms within a regional platform for technical cooperation, technology transfer, research, and training for the regional improvement of antivenom availability, under the coordination of the Pan American Health Organization (PAHO) and its office *Centro Panamericano de Fiebre Aftosa* (Panaftosa).

As part of the ongoing activities of RELAPA, a survey was sent by PAHO/Panaftosa to the institutions integrating this network to assess the situation of antivenom manufacture in these laboratories during the period January 2020 to July 2020, with the goal of analyzing in which ways has antivenom production been affected in this extraordinary year, especially regarding the impact of the Coronavirus Disease 2019 (COVID-19) pandemic, which has profoundly stricken Latin America [7].

The survey was sent to the directors of the institutions of RELAPA (the list of institutions is detailed in Fan and colleagues [6]). The survey included the following aspects: (a) What was the demand and the production of antivenoms (including snake, scorpion, spider, and caterpillar antivenoms) during the period January 2020 to July 2020? (b) What was the effect of the COVID-19 pandemic in (i) the number of professional and technical staff working to manufacture antivenom, (ii) acquisitions of consumables and laboratory equipment, (iii) overall budget devoted to antivenom manufacture, and (iv) attention to the COVID-19 crisis in terms of development of therapeutic equine preparations against Severe Acute Respiratory Syndrome Coronavirus 2 (SARS-CoV-2), preparation of diagnostic reagents, quality control activities, or assignment of staff to attend other pandemic issues? (c) Which are the priorities of the laboratories in the near future regarding regional cooperation in the field of antivenom manufacture and quality control? The survey was carried out between October 1 and October 19, 2020, using the platform Qualtrics (Qualtrics XM Platform, Seattle, Washington, United States of America).

The responses to the survey are summarized as follows:

- A total of 357,266 vials of antivenoms were produced by these laboratories in this period. Seven laboratories manufactured antivenoms, whereas 5 laboratories did not. The reasons for halting the production in these cases were the need to carry out improvements in infrastructure for fulfilling the requirements of Good Manufacturing Practices (GMPs) in 4 cases and the restrictions to do face-to-face work in one institution. When compared to the same period during 2019, the volume of production increased in 2 laboratories, remained the same in 6 laboratories, and was reduced in 4 laboratories. An estimated average production of antivenoms in these institutions during the past years (adjusted for 6 months) was approximately 700,000 vials, based on the information provided by Fan and colleagues [6]. Hence, an overall reduction was observed for the region during the first half of 2020.
- Regarding the national demand of antivenom by national public health institutions, as compared to the same period in 2019, the demand increased for 3 laboratories, remained stable for 7 laboratories, and decreased for 2 of them.
- The laboratories attended various aspects related to the COVID-19 pandemics, as follows: (i) production of therapeutic equine-derived antibodies against SARS-CoV-2 (6 laboratories); (ii) production of diagnostic kits (2 laboratories); (iii) quality control activities (4 laboratories); and (iv) transfer of staff to other departments to attend issues related to the pandemics (7 laboratories). Two laboratories did not attend the COVID-19 emergency.
- Regarding the impact of COVID-19 on the antivenom manufacturing activities, 10 laboratories reported a drop in the personnel dedicated to antivenoms, 7 laboratories had a reduction in the acquisition of consumables for antivenom production, and 4 laboratories reported a reduction in the overall budget assigned to antivenom manufacture.
- In terms of the priority needs of the laboratories for the near future, the following aspects were identified in the survey: (i) implementation of a study on the preclinical efficacy of antivenoms distributed in Latin America; (ii) training of technical and professional staff in aspects related to antivenom production and quality control and implementation of GMPs; (iii) improvements in immunization schemes and innovations in the use of adjuvants; (iv) analysis of the situation of infrastructure and equipment in the laboratories; (v) pharmacovigilance of antivenoms; and (vi) translation to Spanish and Portuguese and distribution of the WHO guidelines for antivenom production and quality control [2].

The main conclusion from this survey is that, despite the crisis generated in the region by the COVID-19 pandemic, which has severely affected Latin America in terms of morbidity, mortality, and socioeconomic impact, the universe of public antivenom manufacturing laboratories has sustained its activity, albeit at a reduced level as compared to historical records [6]. Six laboratories maintained the production of 2019 and 2 increased their output, while 4 reported to have decreased their production. The drop in the output of antivenoms, as compared to the data provided by Fan and colleagues [6], was mostly because several laboratories halted their production during the first half of 2020 for the reasons outlined above. Overall, the survey underscored the resilience of this regional collective of antivenom manufacturers in the context of the COVID-19 crisis while, at the same time, highlights the need to complete the improvements in various laboratories to resume the manufacture of antivenom once the infrastructure works are finished. Whether the fiscal crisis generated in these countries by the pandemics will delay these projects is unknown at present.

It is noteworthy that the national demands of antivenoms by public health authorities (ministries of health and social security systems) in the countries where these manufacturers are located was reduced only in 2 cases, as compared to the same period in 2019. This suggests that public health authorities in the region have not neglected the relevance of snake bite envenoming, even during this unprecedented health and fiscal crisis. Whether this trend will continue is unknown, but it is expected that envenomings by animal bites and stings will receive the required attention by regional and national health authorities in Latin America in the coming years.

It is of interest that 10 out of 12 institutions where antivenoms are manufactured attended in various ways the COVID-19 emergency. In particular, the regional efforts to generate horse-derived immunoglobulin preparations anti-SARS-CoV-2 for the treatment of the coronavirus infections are remarkable and derive from the long-standing regional tradition in the development and manufacture of antivenoms.

Finally, the survey identified a series or priority actions required to improve the landscape of antivenom manufacture, control, and availability in the region. On the basis of this list of priorities, activities are being planned for the year 2021, including (i) the preparation of a research project to assess the preclinical efficacy of antivenoms, (ii) the translation of the WHO guidelines of antivenoms, together with the organization of remote teaching modules on the different aspects of these guidelines, to be offered to the laboratories of RELAPA, and (iii) the implementation of a technical consultation virtual platform for exchanges of information and expertise among RELAPA members in issues related to antivenom production. It is expected that these actions will strengthen the regional capacity for antivenom manufacture and control, with the consequent impact in the availability of this life-saving drug in Latin America.
